# Magnitude of the Placebo Response Across Treatment Modalities Used for Treatment-Resistant Depression in Adults: A Systematic Review and Meta-analysis

**DOI:** 10.1001/jamanetworkopen.2021.25531

**Published:** 2021-09-24

**Authors:** Brett D. M. Jones, Lais B. Razza, Cory R. Weissman, Jewel Karbi, Tya Vine, Louise S. Mulsant, Andre R. Brunoni, M. Ishrat Husain, Benoit H. Mulsant, Daniel M. Blumberger, Zafiris J. Daskalakis

**Affiliations:** 1 Department of Psychiatry, University of Toronto, Toronto, Ontario, Canada; 2 Department of Internal Medicine, Faculty of Medicine, University of Sao Paulo, Sao Paulo, Brazil; 3 Laboratory of Neurosciences, Instituto Nacional de Biomarcadores em Neuropsiquiatria, Department and Institute of Psychiatry, Faculdade de Medicina, University of Sao Paulo, Sao Paulo, Brazil; 4 Centre for Addiction and Mental Health, Toronto, Ontario, Canada; 5 Michael G. Degroote School of Medicine, McMaster University, Hamilton, Ontario, Canada; 6 Nova Scotia Health, Halifax, Nova Scotia, Canada; 7 Department of Psychiatry, University of California San Diego

## Abstract

**Importance:**

The placebo effect in depression clinical trials is a substantial factor associated with failure to establish efficacy of novel and repurposed treatments. However, the magnitude of the placebo effect and whether it differs across treatment modalities in treatment-resistant depression (TRD) is unclear.

**Objective:**

To examine the magnitude of the placebo effect in patients with TRD across different treatment modalities and its possible moderators.

**Data Sources:**

Searches were conducted on MEDLINE, Web of Science, and PsychInfo from inception to June 21, 2021.

**Study Selection:**

Randomized clinical trials (RCTs) were included if they recruited patients with TRD and randomized them to a placebo or sham arm and a pharmacotherapy, brain stimulation, or psychotherapy arm.

**Data Extraction and Synthesis:**

Independent reviewers used standard forms for data extraction and quality assessment. Random-effects analyses and standard pairwise meta-analyses were performed.

**Main Outcomes and Measures:**

The primary outcome was the Hedges *g* value for the reported depression scales. Secondary outcomes included moderators assessed via meta-regression and response and remission rates. Heterogeneity was assessed with the *I*^2^ test, and publication bias was evaluated using the Egger test and a funnel plot. Cochrane Risk of Bias Tool was used to estimate risks.

**Results:**

Fifty RCTs were included involving various types of placebo or sham interventions with a total of 3228 participants (mean [SD] age, 45.8 [6.0] years; 1769 [54.8%] female). The pooled placebo effect size for all modalities was large (*g* = 1.05; 95% CI, 0.91-1.1); the placebo effect size in RCTs of specific treatment modalities did not significantly differ. Similarly, response and remission rates associated with placebo were comparable across modalities. Heterogeneity was large. Three variables were associated with a larger placebo effect size: open-label prospective treatment before double-blind placebo randomization (β = 0.35; 95% CI, 0.11 to 0.59; *P* = .004), later year of publication (β = 0.03; 95% CI, 0.003 to 0.05; *P* = .03), and industry-sponsored trials (β = 0.34; 95% CI, 0.09 to 0.58; *P* = .007). The number of failed interventions was associated with the probability a smaller placebo effect size (β = −0.12; 95% CI, −0.23 to −0.01, *P* = .03). The Egger test result was not significant for small studies’ effects.

**Conclusions and Relevance:**

This analysis may provide a benchmark for past and future clinical RCTs that recruit patients with TRD standardizing an expected placebo effect size.

## Key Points

**Question:**

What is the placebo effect magnitude in different treatment modalities used for management of patients with treatment-resistant depression?

**Findings:**

In this systematic review and meta-analysis of 3228 patients with treatment-resistant depression in 50 randomized clinical trials, the placebo effect size was large and consistent across treatment modalities. Response and remission rates associated with placebo effect were comparable across modalities.

**Meaning:**

The findings of this study suggest a placebo effect size benchmark may be used to interpret the findings of past and future clinical trials.

## Teaser

This systematic review and meta-analysis examines the placebo effect shown in randomized clinical trials of patients with treatment-resistant depression.

## Introduction

Major depressive disorder (MDD) is a relapsing and remitting illness, and many patients do not respond to available treatments.^1,2^ The standard to evaluate a new intervention for MDD uses a randomized clinical trial (RCT) with placebo as the control design that can distinguish the benefit of an active treatment compared with the nonspecific benefit of the placebo response. The placebo response is defined as the therapeutic effect produced by a placebo or sham intervention that is not due to any inherent properties of the placebo. Several novel or repurposed treatments have not been able to establish efficacy in the context of large placebo effects.^3^ This phenomenon is a challenge for researchers; however, attention has begun to focus on trying to understand and quantify the magnitude of the placebo response.^1,2^

In RCTs of non–treatment-resistant depression (non-TRD) in patients with MDD,^4,5,6,7,8,9,10,11,12,13,14^ the placebo effect has been found to have a large magnitude and to be associated with several factors, although with some inconsistent findings. These factors have included later publication years, number of trial arms, multicenter setting, dosing schedule, increased length of the trial, sham device placement, the magnitude of active response, early score fluctuations, and inflation of baseline severity.^4,5,6,7,8,9,10,11,12,13,14^ A large meta-analysis evaluating the placebo effect in depression (256 RCTs; n = 26 324) found placebo-response rates of about 35% to 40%.^13^ However, this analysis included only RCTs of antidepressant drugs in patients without TRD.^13^ Treatment-resistant depression is commonly defined as the lack of response to 2 separate antidepressant trials of adequate dose and duration. Previous meta-analyses have suggested that TRD is associated with a smaller placebo effect than non-TRD (repetitive transcranial magnetic stimulation [rTMS] and escitalopram trials).^15,16^ Nonetheless, to our knowledge, no analysis has assessed the placebo effect in patients with TRD receiving other treatment modalities.

Patients with TRD often receive multiple treatment modalities and have lower rates of response and remission; thus, one would expect them to experience less benefit from the nonspecific effects of treatment. This lack of response highlights the importance of quantifying the placebo effect in TRD, how it may differ across treatment modalities, and what may contribute to it. An appreciation of the expected placebo effect of specific treatment modalities in TRD would provide a benchmark to inform interpretation of past and future RCTs. We therefore conducted a systematic review and meta-analysis to quantify the placebo effect across treatment modalities in TRD. We explored the methodological, demographic, and clinical variables that may contribute to placebo effect. This quantitative analysis is needed to interpret previous RCTs and emerging treatments as well as potentially identifying the beneficial aspect of the placebo effect.

## Methods

**Figure 1.**
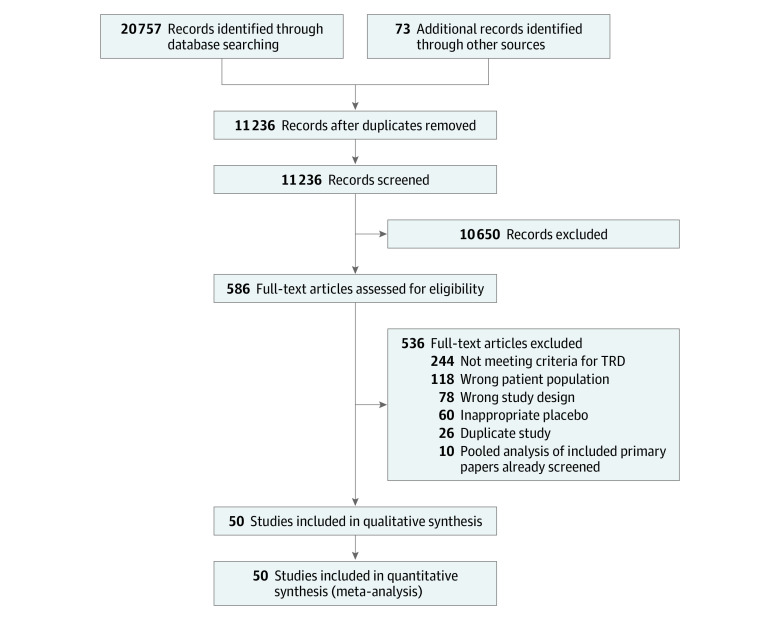
Preferred Reporting Items for Systematic Reviews and Meta-analyses Flow Diagram for Inclusion of Studies

Searches were conducted on MEDLINE, Web of Science, and PsychInfo from inception to June 21, 2021 (eAppendix 2 in the Supplement). In addition, references from relevant reviews were searched.^15,17,18,19,20,21^ Figure 1 provides an overview of the number of studies screened and full texts reviewed. This study followed the Preferred Reporting Items for Systematic Reviews and Meta-analyses (PRISMA) reporting guideline. The protocol was developed a priori and was published and registered^22,23^ (trial registration: PROSPERO Identifier: CRD42020190465).

Study designs were limited to parallel-arm, double-blind placebo-controlled RCTs or the first phase of randomized crossover trials that exclusively recruited patients with TRD and randomized them to at least 1 placebo arm, as specified in the protocol.^22^ Included active interventions were pharmacologic and somatic treatments (Maudsley Treatment Inventory) and psychotherapeutic interventions from the National Institute for Health and Care Excellence (NICE) guidelines.^24,25^ A placebo arm was defined as an inert pill placebo, parenteral placebo, sham device, or sham therapy that does not include any theoretical active property to induce the proposed therapeutic effect. Wait list or treatment as usual were not included as placebo interventions. Randomized clinical trials that used an open-label prospective antidepressant treatment phase to establish treatment resistance before randomization were included. Exclusion criteria were studies in which more than 15% of participants had bipolar depression, included patients with other psychiatric disorders, had sample sizes of less than 10 participants (5 per treatment arm), or used a noninert placebo arm.

Data were extracted by 3 of us (L.B.R., J.K., L.S.M.) and discrepancies were resolved by consensus. Data were confirmed by 1 of us (B.D.M.J.). Variables extracted included demographic characteristics, methodologic data, clinical/outcome data, and placebo-specific data. The demographic characteristics were age and sex. Methodological data included treatment strategy (augmentation vs monotherapy), multicenter setting, length of the trial (calculated as number of days for double-blind treatment), number of treatment arms, year of publication, trial design (ie, whether the trial used open-label prospective treatment to determine treatment resistance before double-blind randomization), and whether the trial was industry sponsored (defined as being sponsored by a pharmaceutical company or a medical device manufacturer). Randomized clinical trials funded by the US Department of Veterans Affairs; government agencies, such as the Canadian Institutes of Health Research or National Institute of Mental Health; nonprofit foundations, such as National Alliance for Research on Schizophrenia & Depression, or Brain & Behavior Research Foundation; or hospitals or universities were not considered to be industry sponsored. Clinical/outcome data comprised treatment modality, sample size, mean (SD) scores of depression rating scales at baseline and end point in both the placebo and active groups, number of responders/remitters (as defined by the individual studies), number of failed antidepressant trials, past depressive episodes, and length of the current episode. Placebo-specific data included route (eg, oral vs parenteral) of administration and number of days receiving placebo. This variable was calculated by determining how many days during the trial period participants would receive placebo or sham intervention.

If the mean (SD) scores of the depression rating scales were not reported in the study, the corresponding author was contacted by email. If no reply was obtained, we extracted the data from the study graphs. Moreover, when only the SD of the change was reported, we imputed the data as recommended by the Cochrane group.^26^ For studies that included an open-label prospective (run-in) treatment phase, effect size was calculated from the start of the double-blind randomized phase.

The quality of the studies was assessed independently by 2 of us (B.D.M.J. and C.R.W.) and recorded using the Cochrane Risk of Bias tool.^27^ Thus, 5 bias domains were assessed: selection (randomization and allocation concealment), performance (blinding of participants and personnel), attrition (incomplete outcome data), detection (blinding of outcome assessment), and reporting (selective outcome reporting). For a judgment of the overall risk of bias, we followed the Cochrane recommendation: low risk of bias (low risk for all domains or some concerns of bias for 1 domain), unclear risk of bias (2 or 3 domains were rated as presenting some cause for concern), and high risk of bias (>3 domains with some bias concerns and/or ≥1domain with high risk of bias).

The analyses were performed using Stata, version 17 (StataCorp LLC) software. Considering that most studies have small samples, we used the Hedges *g* to estimate the effect size of the placebo response. The effect size was computed based on the baseline and end point sample sizes and means (SDs) of the primary depression score scales of the placebo group of each study. A random-effects model (restricted maximum-likelihood method instead of fixed-effect models was used considering that study heterogeneity would be high). The model provides wider 95% CIs compared with fixed-effects models, thereby providing a more conservative estimation of summary effect size.^28^ The model assumes that the effect sizes of the studies are different from each other and represent a random sample from a larger population of studies.^29^ The components of the random-effects model applied in this meta-analysis are described in eAppendix 1 in the Supplement. Heterogeneity among studies was assessed with *I^2^* analysis and was considered high when presenting a value greater than or equal to 50% as suggested elsewhere.^30^ Small studies effects were assessed using the Begg modified funnel plot, the Duval and Tweedie trim-and-fill procedure, and the Egger regression intercept. The Egger test was considered significant for small studies’ effects when presenting findings significant at *P* < .05.

Meta-regression and subgroup analyses were applied to explore potential moderators of the placebo effect. To increase overall sample size, treatment modalities were pooled for the meta-regression. Meta-regressions were conducted using only 1 variable at a time. Subgroup analysis was conducted to compare the effect size of the placebo response among treatment modalities. Values were considered statistically significant when presenting findings at *P* < .05 for both meta-regression and subgroup analysis. The mean of the percentage of responders and remitters for each treatment modality was also assessed. In addition, a sensitivity analysis was conducted looking at low risk of bias.

## Results

Our search yielded 11 236 studies that were screened; full texts of 605 articles were reviewed and 50 RCTs (N = 3228) were included.^31,32,33,34,35,36,37,38,39,40,41,42,43,44,45,46,47,48,49,50,51,52,53,54,55,56,57,58,59,60,61,62,63,64,65,66,67,68,69,70,71,72,73,74,75,76,77,78,79,80^ the mean (SD) age of the participants was 45.8 (6.0) years, and the mean (SD) proportion of women was 54.8% (20.7%) (Figure 1 and Table 1). Additional clinical characteristics are described in eTable 3 in the Supplement. Sixteen RCTs were classified to have a low risk of bias; 19, an unclear risk of bias, and 16, a high risk (eTable 1 in the Supplement). No psychotherapy RCTs met our eligibility criteria. Placebo or sham interventions were categorized as pill placebo, liquid placebo (trials in this analysis used ayahuasca, a South American psychoactive brew), parenteral placebo, sham rTMS, sham transcranial direct current stimulation (tDCS), or sham invasive brain stimulation.

**Table 1.** Demographic, Clinical, and Methodological Characteristics of Included Studies in the Systematic Review and Meta-analysis

Source	Demographic characteristics				Methodologic characteristics			Placebo characteristics			Overall risk of bias
	No.	Women No. (%)	Age, mean (SD), y	Depression score	Design	Augmentation	Treatment	Placebo route	Total days receiving placebo	Times placebo was measured	
Bakim et al,^31^ 2012	12	11 (91.7)	44.4 (10.2)	HDRS-17	SC, treatment not prospective, 3 treatment arms, 42 d	Yes	rTMS	Noninvasive brain stimulation	30	6	Unclear
Barbee et al,^32^ 2011	48	33 (68.8)	45.8 (11)	MADRS	MC, prospective treatment, 2 arms, 70 d	Yes	Lamotrigine	Oral	70	6	Low
Bauer et al,^75^ 2019	441	302 (68.5)	46.4 (12.1)	MADRS	MC, prospective treatment, 2 arms, 168 d	Yes	Brexiprazol	Oral	168	9	High
Bennabi et al,^74^ 2015	12	5 (45.5)	59.9 (15.4)	HDRS-21	SC, treatment not prospective, 2 arms, 35 d	Yes	tDCS	Noninvasive brain stimulation	5	3	Unclear
Berman et al,^73^ 2007	176	113 (64.2)	44.2 (10.9)	MADRS	MC, prospective treatment, 2 arms, 42 d	Yes	Aripiprazole	Oral	42	6	Low
Berman et al,^33^ 2009	172	117 (68.0)	45.6 (11.3)	MADRS	MC, prospective treatment, 2 arms, 42 d	Yes	Aripiprazole	Oral	42	6	Unclear
Blumberger et al,^35^ 2012	22	14 (70.0)	45.8 (13.4)	HDRS-17	SC, treatment not prospective, 3 arms, 42 d	Yes	rTMS	Noninvasive brain stimulation	30	6	Low
Blumberger et al,^34^ 2012	11	10 (90.9)	49.7 (9.4)	HDRS-17	SC, treatment not prospective, 2 arms, 21 d	Yes	tDCS	Noninvasive brain stimulation	15	2	Low
Blumberger et al,^36^ 2016	41	24 (59.0)	48.1 (12.0)	HAMD-17	SC, treatment not prospective, 3 arms, 42 d	Yes	rTMS	Noninvasive brain stimulation	30	2	Low
Boutros et al,^37^ 2002	9	1 (11.0)	52 (7)	HDRS-25	SC, treatment not prospective, 2 arms, 14 d	Yes	rTMS	Noninvasive brain stimulation	10	5	Low
Chen et al,^38^ 2013	10	4 (40.0)	47.3 (3.5)	HAMD-17	SC, treatment not prospective, 2 arms, 14 d	Yes	rTMS	Noninvasive brain stimulation	10	2	High
Coenen et al,^72^ 2019	8	3 (37.5)	49.9 (12)	MADRS	SC, treatment not prospective, 2 arms, 56 d	Yes	DBS	Invasive brain stimulation	1	4	High
Concerto et al,^39^ 2015	15	7 (46.7)	53 (6.7)	HDRS	SC, treatment not prospective, 2 arms, 28 d	Yes	rTMS	Noninvasive brain stimulation	20	1	High
Dougherty et al,^40^ 2015	14	5 (35.7)	48.9 (8.9)	MADRS	MC, treatment not prospective, 2 arms, 112 d	Yes	DBS	Invasive brain stimulation	1	6	Low
Fava et al,^77^ 2015	85	56 (65.9)	45.9 (10.6)	MADRS	MC, prospective treatment, 2 arms, 42 d	Yes	nAChR antagonist	Oral	42	5	High
Fava et al,^41^ 2018	81	61 (75.3)	45.2 (10.2)	MADRS	MC, prospective treatment, 3 arms, 56 d	Yes	Cariprazine	Oral	56	5	Low
Fitzgerald et al,^42^ 2012	20	8 (40.0)	44.9 (15.7)	HDRS-17	SC, treatment not prospective, 3 arms, 21 d	Yes	rTMS	Noninvasive brain stimulation	15	1	High
Garcia-Toro et al,^43^ 2001	18	8 (44.4)	50 (11)	HDRS-21	SC, treatment not prospective, 2 arms, 14	Yes	rTMS	Noninvasive brain stimulation	10	2	Unclear
Garcia-Toro et al,^76^ 2006	10	7 (70.0)	47.2 (11.8)	HDRS-21	SC, treatment not prospective, 3 arms, 14	Yes	rTMS	Noninvasive brain stimulation	10	2	Unclear
Heresco-Levy et al,^44^ 2013	13	8 (61.5)	53 (10.2)	HDRS-21	MC, treatment not prospective, 2 arms, 42 d	Yes	D-cycloserine	Oral	42	3	Low
Hobart et al,^45^ 2018	206	144 (71.3)	41.8 (11.7)	MADRS	MC, prospective treatment, 3 arms, 42 d	Yes	Brexiprazol	Oral	42		Low
Hobart et al,^71^ 2018	202	149 (72.3)	42.7 (12.5)	MADRS	MC, prospective treatment, 2 arms, 42	Yes	Brexiprazol	Oral	42	6	Unclear
Holtzheimer et al.^46^ 2004	8	3 (42.9)	45.4 (4.9)	HDRS-17	SC, treatment not prospective, 2 arms 14 d	No	rTMS	Noninvasive brain stimulation	10	2	Unclear
Holtzheimer et al,^70^ 2017	30	17 (57.0)	48.7 (0.6)	MADRS	MC, treatment not prospective, 2 arms, 183 d	Yes	DBS	Invasive brain stimulation	1	2	Low
Husain et al,^69^ 2017	20	11 (55.0)	34.9 (2.5)	HDRS-17	MC, treatment not prospective, 2 arms, 84 d	Yes	MiNocycline	Oral	84	3	Unclear
Ionescu et al,^68^ 2019	13	3 (23.0)	45.3 (11.7)	HAMD-28	SC, treatment not prospective, 2 arms, 21 d	Yes	Ketamine	Parenteral	6	6	Low
Kamijima et al,^66^ 2013	195	80 (41.0)	38.7 (9.2)	MADRS	MC, prospective treatment, 3 arms, 42 d	Yes	Aripiprazole	Oral	42	6	Unclear
Kamijima et al,^67^ 2018	203	72 (35.5)	39.5 (11.8)	MADRS	MC, prospective treatment, 2 arms, 42 d	Yes	Aripiprazole	Oral	42	6	Low
Kauffmann et al,^47^ 2004	5	4 (91.7)	51.7 (17.2)	HDRS-21	SC, treatment not prospective, 2 arms, 14 d	Yes	rTMS	Noninvasive brain stimulation	10	2	Unclear
Li et al,^48^ 2014	15	11 (73.3)	46.9 (NA)	HDRS-17	SC, treatment not prospective, 4 arms, 14 d	Yes	rTMS	Noninvasive brain stimulation	10	2	Unclear
Marcus et al,^49^ 2008	190	128 (67.4)	44.4 (10.7)	MADRS	42	Yes	Aripiprazole	Oral	42	6	High
McAllister-Williams et al,^50^ 2016	82	52 (63.0)	45.2 (10.4)	MADRS	MC, treatment not prospective, 2 arms, 35 d	Yes	Metyrapone	Oral	21	2	Low
Chen et al,^65^ 2019	16	13 (81.3)	49.9 (8.1)	MADRS	SC, treatment not prospective, 3 arms, 3 d	Yes	Ketamine	Parenteral	1	1	High
Nierenberg et al,^64^ 2003	17	7 (41.2)	39.7 (11.9)	HSRD-17	42	Yes	Lithium	Oral	42	3	Unclear
Palhano-Fontes et al.^63^ 2019	15	10 (66.7)	44.2 (12.0)	HDRS-17	SC, treatment not prospective, 2 arms, 7 d	No	Ayahuasca	Oral	1	3	Unclear
Pallanti et al,^51^ 2010	20	12 (60.0)	47.9 (9.1)	HDRS-17	SC, treatment not prospective, 3 arms, 21 d	Yes	rTMS	Noninvasive brain stimulation	15	3	High
Santos et al,^52^ 2008	17	11 (65.0)	29 (NA)	MADRS	SC, treatment not prospective, 2 arms, 56 d	Yes	Lamotrigine	Oral	56	4	High
Shelton et al,^53^ 2001	10	NA	NA	MADRS	SC, prospective treatment, 3 arms, 56 d	Yes	Olanzapine	Oral	56	8	High
Su et al,^62^ 2017	24	15 (62.5)	48.6 (8.2)	HDRS-17	SC, treatment not prospective, 3 arms, 5 d	Yes	Ketamine	Parenteral	1	8	Unclear
Thase et al,^54^ 2015	191	137 (71.6)	45.2 (11.3)	MADRS	MC, prospective treatment, 2 arms, 42 d	Yes	Brexpiprazole	Oral	42	6	High
Thase et al,^55^ 2015	221	146 (66.1)	46.6 (11)	MADRS	MC, prospective treatment, 3 arms, 42 d	Yes	Brexpiprazole	Oral	42	6	High
Theleritis et al,^56^ 2017	20	10 (50)	38 (9.9)	HDRS-17	SC, treatment not prospective, 4 arms, 21 d	No	rTMS	Noninvasive brain stimulation	15	3	Unclear
Theleritis et al,^56^ 2017	24	10 (31.6)	39.4 (8.9)	HDRS-17	SC, treatment not prospective, 4 arms, 21 d	No	rTMS	Noninvasive brain stimulation	15	3	Unclear
Triggs et al,^57^ 2010 (left sham)	7	2 (28.6)	41.9 (14.1)	HAMD-24	SC, treatment not prospective, 4 arms, 14 d	Yes	rTMS	Noninvasive brain stimulation	10	2	Unclear
Triggs et al,^57^ 2010 (right sham)	7	4 (57.1)	46.6 (20.2)	HAMD-24	SC, treatment not prospective, 4 arms, 14 d	Yes	rTMS	Invasive Brain Stimulation	10	2	Unclear
Yesavage et al,^61^ 2018	83	18 (21.7)	54.8 (12.6)	HSRD-24	MC, treatment not prospective, 2 arms, 42 d	Yes	rTMS	Noninvasive brain stimulation	30	3	Low
Zheng et al,^60^ 2010	15	5 (33.3)	26.7 (4.3)	HAMD-17	SC, treatment not prospective, 2 arms, 28 d	Yes	rTMS	Noninvasive brain stimulation	20	1	High
Palm et al,^59^ 2012	11	8 (72)	58 (12)	HAMD-24	SC, treatment not prospective, 2 arms, 14 d	Yes	tCDS	Noninvasive brain stimulation	10	2	Low
van Eijndhoven et al,^58^ 2020	16	13 (81)	49.7 (11)	HDRS-17	SC, treatment not prospective, 2 arms, 20	Yes	rTMS	Noninvasive brain stimulation	35	5	Unclear
Rush et al,^79^ 2005	110	73 (66)	45.9 (9)	HAMD-24	MC, treatment not prospective, 2 arms, 70 d	Yes	Vagus nerve stimulation	Invasive brain stimulation	70	7	High
Baeken et al,^80^ 2013	11	5 (45)	47.3 (13.6)	HDRS	SC, treatment not prospective, 2 arms, 7 d	No	rTMS	Noninvasive brain stimulation	4	1	High
Padberg et al,^78^ 1999	6	4 (66.6)	43.3 (11.6)	HDRS	SC, treatment not prospective, 3 arms, 5 d	Yes	rTMS	Noninvasive brain stimulation	5	2	Unclear

Abbreviations: DBS, deep brain stimulation; HDRS, Hamilton Depression Rating Scale; MADRS, Montgomery-Åsberg Depression Rating Scale; MC, multicenter; SC, single-center; rTMS, repetitive transcranial magnetic stimulation; tDCS, transcranial direct current stimulation.

**Figure 2.**
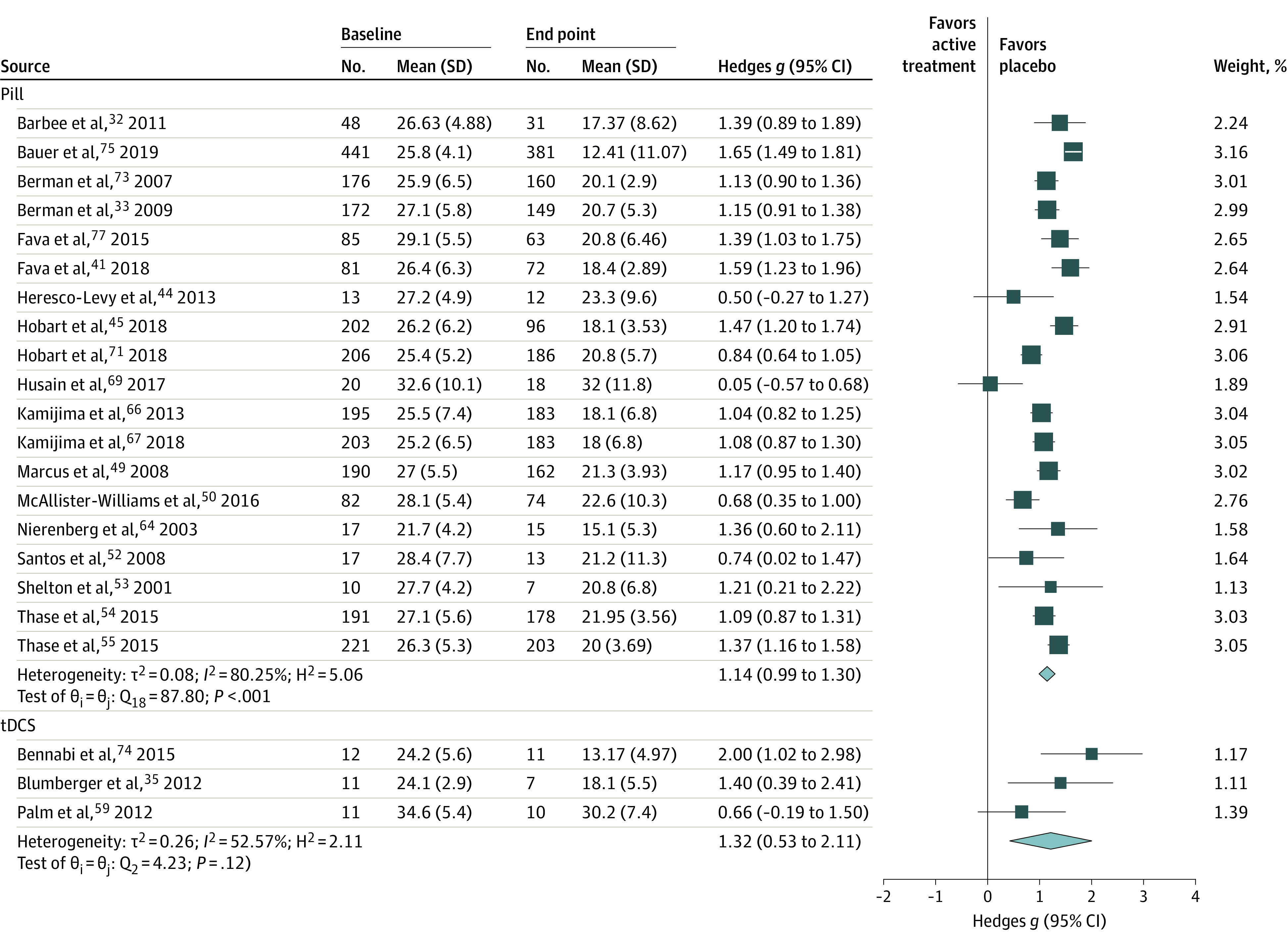
Main Placebo Effect Size of Included Pill and tDCS Studies Studies were grouped by treatment modality and pooled. tDCS indicates transcranial direct current stimulation.

**Figure 3.**
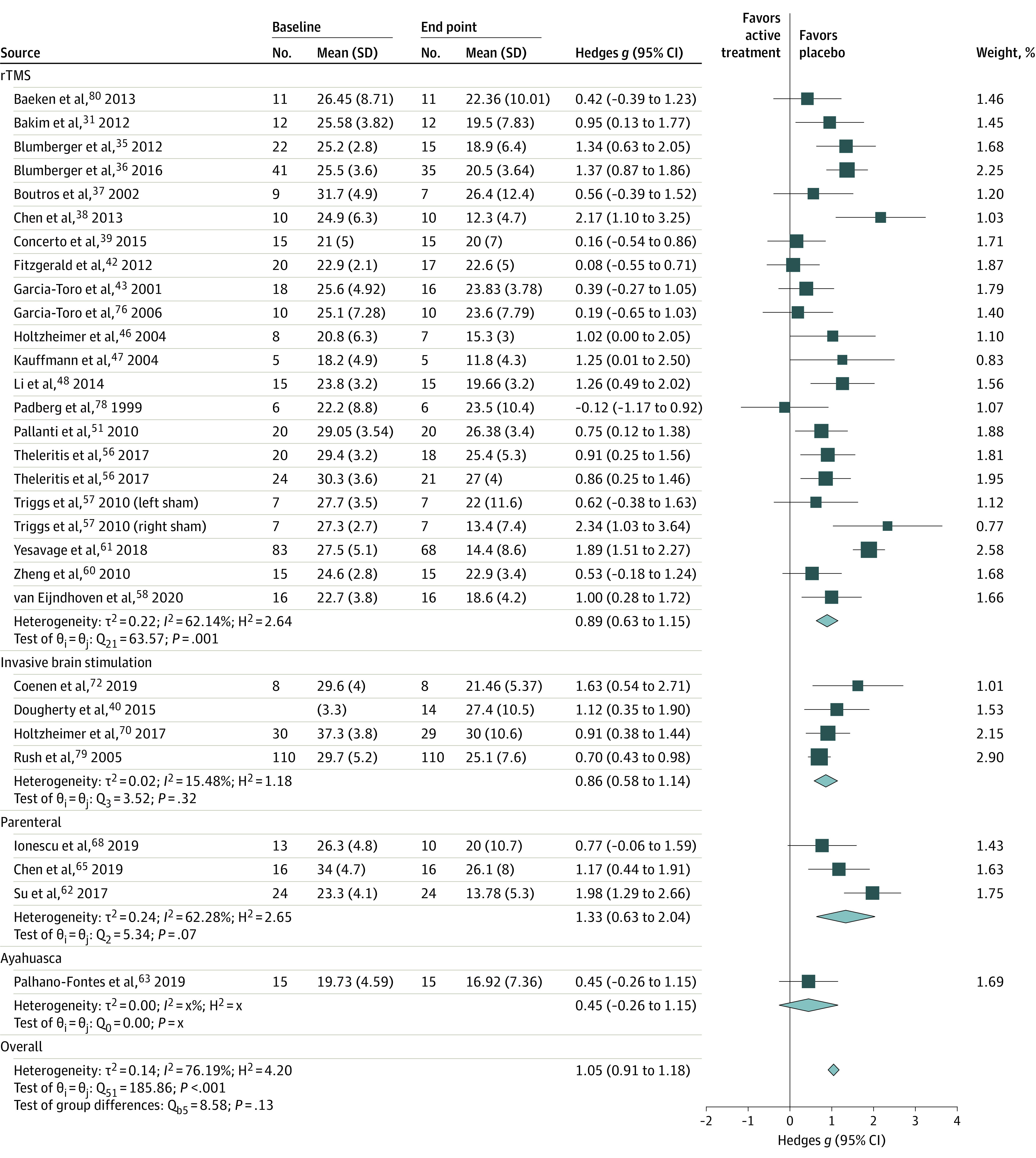
Main Placebo Effect Size of Included rTMS, Invasive Brain Stimulation, Parenteral, Ayahuasca, and Overall Studies Studies were grouped by treatment modality and pooled. Ayahuasca is a South American psychoactive brew. rTMS indicates repetitive transcranial magnetic stimulation.

Studies were pooled based on treatment modality to maintain sufficient similarity in the pairwise analysis. The effect sizes for each treatment modality were pill placebo, *g* = 1.14 (95% CI, 0.99 to 1.30; *I^2^* =  80.25%); parenteral placebo, *g* = 1.33 (95% CI, 0.63 to 2.04; *I^2^* = 62.28%); liquid placebo, *g* = 0.45 (95% CI, −0.26 to 1.15; *I^2^* = 0%); rTMS, *g* = 0.89 (95% CI, 0.63 to 1.15; *I^2^* = 62.14%); tDCS, *g* = 1.32 (95% CI, 0.53 to 2.11; *I^2^* = 52.57%); and invasive brain stimulation, *g* = 0.86 (95% CI, 0.58 to 1.14; *I^2^* = 15.48%). The pooled effect size for all treatment modalities was *g* = 1.05 (95% CI, 0.91 to 1.18) with an overall *I^2^* = 76.19% (Figure 2 and Figure 3).

The funnel plot of the primary outcome showed a symmetrical distribution among the included studies (eFigure 1 in the Supplement). The Egger test corroborated this finding (*z* = 1.18; *P* = .23). We also applied the trim-and-fill method to deeply investigate any asymmetry of the funnel plot (eFigure 2 in the Supplement) and its analysis showed the presence of 5 unpublished studies. Considering these studies in the pooled analyses, the overall effect size was adjusted to *g* = 1.14 (95% CI, 1.00 to 1.29). The overall placebo effect size remained large when considering only studies with a low risk of bias (*g* = 1.18; 95% CI, 0.98 to 1.38) (eFigure 3 in the Supplement).

For a subset of RCTs that reported response rates (n = 42) and remission rates (n = 25), the pooled mean (SD) response rate was 21.2% (14.6%) and the pooled remission rate was 13.0% (9.05%). Modality-specific response and remission rates are described in Table 2.

**Table 2.** Modality-Specific Response and Remission Rates

Modality	Response		Remission	
	No. of studies	% Rate (SD)	No. of studies	% Rate (SD)
Pill	17	21.9 (8.2)	13	16.6 (6.1)
tDCS	2	18.2 (12.8)	2	4.5 (6.4)
rTMS	16	20.8 (21.4)	7	11.2 (13.2)
Invasive brain stimulation	4	19.1 (11.0)	1	7.0 (0)
Parenteral	2	22.8 (14.5)	1	8 (0)
Liquid (Ayahuasca)	1	27 (0)	1	7 (0)

Abbreviations: rTMS, repetitive transcranial magnetic stimulation; tDCS, transcranial direct current stimulation.

Pooled meta-regression analysis revealed that industry-sponsored studies (β = 0.34; 95% CI, 0.09 to 0.59; *P* = .007), year of publication (β = 0.03; 95% CI, 0.003 to 0.05; *P* = .03), and studies that used an open-label prospective treatment phase before double-blind randomization (β = 0.35; 95% CI, 0.11 to 0.59; *P* = .004) had a significantly higher placebo effect. The number of failed interventions during the current episode was associated with a smaller placebo effect (β = −0.12; 95% CI, −0.23 to −0.01; *P* = .03) (Table 3).

**Table 3.** Meta-regression for Placebo Effect

Variable	No.	Coefficient β (95% CI)	*P* value
Publication year	52	0.03 (0.003 to 0.05)	.03
No. of treatment arms	52	0.02 (−0.20 to 0.23)	.89
Prospective treatment	52	0.35 (0.11 to 0.59)	.004
Industry sponsored	52	0.34 (0.09 to 0.58)	.007
Baseline depression severity	52	−0.006 (−0.04 to 0.03)	.74
Total study length, d	52	0.002 (−0.001 to 0.006)	.17
Total days of placebo	52	0.002 (−0.001 to 0.005)	.22
Multicenter	52	0.21 (−0.05 to 0.46)	.11
Augmentation treatment	52	0.35 (−0.13 to 0.83)	.15
Age	51	0.02 (−0.001 to 0.05)	.06
Female %	52	0.001 (−0.006 to 0.009)	.72
Length of current episode	32	0.0003 (−0.004 to 0.005)	.90
Past episodes	24	−0.03 (−0.15 to 0.1)	.68
Effect size of active arm	51	0.08 (−0.08 to 0.25)	.31
No. of failed trials	17	−0.12 (−0.23 to −0.01)	.03

Subgroup analysis found no significant differences in placebo effect among treatment modalities (eTable 2 in the Supplement). Owing to the high heterogeneity between studies, no other subgroup analysis was completed.

## Discussion

In this meta-analysis, we report on the placebo effect in TRD across multiple treatment modalities. We synthesized data from 50 RCTs including 3228 participants who received either pill placebo, parenteral placebo, liquid placebo, sham rTMS, sham tDCS, or sham invasive brain stimulation. The combined placebo effect size for all interventions was large (*g* = 1.05) and the placebo effect sizes for each treatment modality did not significantly differ. This finding is consistent with prior analyses that have shown that placebo in MDD (non-TRD) has a large effect size, seen with RCTs of antidepressants (Cohen *d* = 1.69), rTMS (*g* = 0.8), or tDCS (*g* = 1.09^14,15,81^). Studies in other psychiatric populations have also shown a high placebo effect size, such as negative symptoms in schizophrenia (Cohen *d* = 2.91) and response rate (39.2%) in bipolar depression.^82,83^ Although all of these analyses reported a large effect size, the effect size found in RCTs involving patients with TRD is numerically smaller for pill placebo and numerically larger for sham stimulation than the effect sizes reported in these RCTs involving patients without TRD.^14,15,81^

In our secondary analysis, we assessed response and remission rates as reported in the individual RCTs. The pooled response rate across all treatment modalities was 23.5% and the remission rate was 15.5%. These low rates did not differ significantly across treatment modalities. A large meta-analysis in patients without TRD reported a response rate of placebo to be 35% to 40%, which is numerically higher than our rate of 21.2%.^13^ Another meta-analysis reported a remission rate of 22%, which is numerically higher than our finding of 13.0%.^84^ Earlier studies of placebo effect that have included participants with TRD have found that TRD was associated with a lower placebo response, which may explain why our response and remission rates appear to be lower than in studies involving patients without TRD.^15^

Our meta-regression found that RCTs that used an open-label prospective treatment phase before double-blind randomization, a more recent year of publication, and those that were industry sponsored had a larger placebo effect. A meta-analysis of placebo response in negative symptoms of schizophrenia also reported that placebo response was higher in industry-sponsored clinical trials (Cohen *d* = 6.72) compared with academic-funded trials (Cohen *d* = 1.01).^82^ Only pill placebo RCTs used an open-label prospective treatment phase and only 1 small rTMS RCT was industry sponsored. Previous research has also suggested that, in populations without treatment-resistant MDD, a higher placebo response was associated with more recent year of publication.^85^ Other studies have suggested that finding may reflect methodological changes, such as an increased number of multicenter studies.^86^ Placebo effect has been shown to be associated with expectancy of active treatment and increased activity in reward circuitry.^6,87^ Industry-sponsored RCTs are often investigating novel agents, which may lead to increased expectations of efficacy by participants, although we are unable to confirm this possibility with available data. Another potential contributing factor to the higher placebo effect in the prospective open-label treatment trials is that of delayed antidepressant response. In these studies, patients are continuing the recently started antidepressant from the prospective open-label treatment phase in combination with placebo or another active agent, which may be contributing to a delayed response. The number of failed trials was also significantly associated with a smaller placebo effect. Although only 17 of 50 studies reported on this variable, this finding is consistent with previous literature showing that a higher level of treatment resistance is associated with a smaller placebo effect.^15^

Our analysis did not find that the placebo effect was significantly different across treatment modalities. This finding is consistent with trials comparing rTMS with escitalopram.^16^ The large placebo effect in TRD does not change substantially based on modality. This lack of effect supports the notion that the nonspecific factors contributing to the placebo effect in TRD are prevalent in any clinical trial. Our findings suggest that the invasiveness of the intervention (eg, pill vs invasive brain stimulation) does not substantially affect the placebo effect in TRD. Given the consistency of the placebo effect across treatment modalities in TRD, neurobiological and common psychological factors need to be further investigated.

Our primary finding that the placebo effect in TRD is large (*g* = 1.05) and that it is consistent across treatment modalities may help to interpret the results of past and future RCTs. Researchers who conduct clinical trials may now compare their results with a benchmark for expected placebo effect in TRD. For instance, a placebo-controlled RCT that reports negative findings but had a placebo effect size greater than *g* = 1.05 could be interpreted as a false-negative; conversely, an RCT that reports positive findings with a placebo effect size less than *g* = 1.05 could be a false-positive. Our results also provide a context for noninferiority trials that do not use a placebo or sham control intervention. Treatments that differentiate from this benchmark (*g* = 1.05) by a predetermined margin would be expected to be superior to placebo.

### Limitations

This study has limitations. Although we were able to include several different treatment modalities in our analysis, no psychotherapy RCTs met our eligibility criteria. Psychotherapy trials in TRD were either open-label, single arm, had an active comparator, or used treatment as usual or a wait-list condition. There were also no RCTs of electroconvulsive therapy or magnetic seizure therapy that used sham versions of these procedures in a TRD population. Another limitation is the definition of TRD. Although we used the most common definition, there is no standard TRD definition. A large proportion of studies that were excluded reported including patients with TRD even though they defined TRD based on only one failed antidepressant trial. For this reason, we were unable to include 2 large industry-sponsored trials for rTMS.^88,89^ Future research would benefit from an improved and consistent definition of TRD. Current definitions of TRD are homogeneous and do not consistently account for a number of important factors, such as specific treatments failed (eg, psychotherapy vs brain stimulation vs selective serotonin reuptake inhibitor vs serotonin and norepinephrine reuptake inhibitor) and psychosocial factors. Although we aimed to compare the placebo effect across treatment modalities, our results are limited by the absence of direct comparison of 2 placebo modalities (ie, sham brain stimulation vs pill placebo).

## Conclusions

The present systematic review and meta-analysis compared the placebo effect in TRD across different treatment modalities. Our main finding was that the placebo effect in TRD appears to be large and consistent across treatment modalities. The effect size in the studies included in our analysis (*g* = 1.05) may serve as a benchmark to assess placebo effect in future TRD RCTs. Factors that increase the placebo effect appear to include using an open-label, prospective treatment phase and industry sponsorship. To better understand the placebo effect, the following improvements are needed: more consistent reporting of data, an agreement on a standard definition of TRD and its possible subgroups, and further assessment and reporting of participants’ expectations and experiences within a clinical trial.

## Notes

Supplement. **eTable 1.** Risk of Bias **eTable 2.** Subgroup Analysis of Treatment Modalities **eTable 3.** Clinical Characteristics **eAppendix 1.** Components of the Randomized Effect Model Used for Our Analyses **eAppendix 2.** Search Strategy **eFigure 1.** Funnel Plot of Included Studies **eFigure 2.** Trim and Fill Funnel Plot **eFigure 3.** Forest Plot of Main Placebo Effect Size Grouped by Risk of Bias
