# Error in Figure 2 and Figure 3

**DOI:** 10.1001/jamanetworkopen.2021.44993

**Published:** 2021-12-30

In the Original Investigation titled “Magnitude of the Placebo Response Across Treatment Modalities Used for Treatment-Resistant Depression in Adults: A Systematic Review and Meta-analysis,”^1^ published September 24, 2021, there was an error in Figure 2 and Figure 3. The labels above the plots showing data points and error bars indicating Hedges *g* values were incorrect and should not have been included in the figures. This article has been corrected.^1^
