# Supplementary material for: Magnitude of the Placebo Response Across Treatment Modalities Used for Treatment-Resistant Depression in Adults: A Systematic Review and Meta-analysis
Source: JAMA Netw Open. 2021 Sep 24;4(9):e2125531. doi: 10.1001/jamanetworkopen.2021.25531 (PMC8463940; doi:10.1001/jamanetworkopen.2021.25531)
Supplement: Supplement. — eTable 1. Risk of Bias eTable 2. Subgroup Analysis of Treatment Modalities eTable 3. Clinical Characteristics eAppendix 1. Components of the Randomized Effect Model Used for Our Analyses eAppendix 2. Search Strategy eFigure 1. Funnel Plot of Included Studies eFigure 2. Trim and Fill Funnel Plot eFigure 3. Forest Plot of Main Placebo Effect Size Grouped by Risk of Bias [file jamanetwopen-e2125531-s001.pdf]

Supplementary Online Content

Jones BDM, Razza LB, Weissman CR, et al. Magnitude of the placebo response across treatment modalities used for treatment-resistant depression in adults: a systematic review and meta-analysis. *JAMA Netw Open*. 2021;4(9):e2125531. doi:10.1001/jamanetworkopen.2021.25531

eTable 1. Risk of Bias

eTable 2. Subgroup Analysis of Treatment Modalities

eTable 3. Clinical Characteristics

eAppendix 1. Components of the Randomized Effect Model Used for Our Analyses

eAppendix 2. Search Strategy

eFigure 1. Funnel Plot of Included Studies

eFigure 2. Trim and Fill Funnel Plot

eFigure 3. Forest Plot of Main Placebo Effect Size Grouped by Risk of Bias

This supplementary material has been provided by the authors to give readers additional information about their work.

© 2021 Jones BDM et al. *JAMA Network Open*

eTable 1. Risk of Bias

Study	Random sequence generation	Allocation Concealment	Blinding (investigators)	Blinding (participants)	Selective Outcome Reporting	Missing Data	Overall bias
Bakim et al.[31] 2012	1	1	0.5	0.5	1	0.5	unclear
Barbee et al.[32] 2011	1	1	1	1	1	1	low
Bauer et al.[75] 2018	1	1	1	1	1	0	high
Bennabi et al.[74] 2015	1	1	0.5	0.5	1	0.5	unclear
Berman et al.[73] 2007	1	1	1	1	1	1	low
Berman et al.[33] 2009	0.5	0.5	1	1	1	1	unclear
Blumberger et al.[35] 2012	1	1	1	1	1	0.5	low
Blumberger et al.[34] 2012	1	1	1	1	1	1	low
Blumberger et al.[36] 2016	1	1	1	1	1	1	low
Boutros et al.[37] 2002	1	1	1	1	1	0.5	low
Chen et al.[38] 2013	0.5	0.5	0.5	0.5	1	0.5	high
Coenen et al.[72] 2019	0	0	1	1	1	1	high
Concerto et al.[39] 2015	0	0	0.5	1	1	1	high
Dougherty et al.[40] 2015	1	1	1	1	1	0.5	low
Fava et al.[77] 2015	0.5	0.5	0.5	0.5	1	0	high
Fava et al.[41] 2018	1	1	1	1	1	1	low
Fitzgerald et al.[42] 2012	0	0	1	1	1	0.5	high
Garcia-Toro et al.[76] 2001	0.5	0.5	1	1	0.5	1	unclear
Garcia-Toro et al.[43] 2006	0.5	0.5	1	1	0.5	1	unclear

© 2021 Jones BDM et al. *JAMA Network Open*

Study	Random sequence generation	Allocation Concealment	Blinding (investigators)	Blinding (participants)	Selective Outcome Reporting	Missing Data	Overall bias
Heresco-Levy et al.[44] 2012	1	0.5	1	1	1	1	low
Hobart et al.[45] 2018	1	0.5	1	1	1	1	low
Hobart et al.[71] 2018	0.5	1	1	1	1	0.5	unclear
Holtzheimer et al.[46] 2004	0.5	0	0.5	0.5	1	1	unclear
Holtzheimer et al.[70] 2017	1	1	1	1	1	1	low
Husain et al.[69] 2017	1	0.5	1	1	1	0.5	unclear
Ionescu et al.[68] 2019	1	0.5	1	1	1	1	low
Kamijima et al.[66] 2013	0.5	0.5	0.5	1	1	1	unclear
Kamijima et al.[67] 2018	0.5	1	1	1	1	1	low
Kauffmann et al.[47] 2004	0.5	0.5	1	0.5	1	1	unclear
Li et al.[48] 2014	0.5	0.5	1	0.5	1	1	unclear
Marcus et al.[49] 2008	0.5	0.5	0.5	1	1	0.5	high
McAllister-Williams et al.[50] 2015	1	1	1	1	1	0.5	low
Mu-Hong Chen et al.[65] 2019	0.5	0.5	0.5	0.5	1	0.5	high
Nierenberg et al.[64] 2003	0.5	0.5	0.5	1	1	1	unclear
Palhano-Fontes et al.[63] 2019	1	1	0.5	1	1	0.5	unclear
Pallanti et al.[51] 2010	0.5	0.5	0.5	0.5	1	0.5	high
Santos et al.[52] 2008	1	1	1	1	0	0.5	high
Shelton et al.[53] 2001	0.5	0.5	0.5	1	0.5	1	high
Su et al.[62] 2017	0.5	0.5	0.5	1	1	1	unclear

© 2021 Jones BDM et al. *JAMA Network Open*

Study	Random sequence generation	Allocation Concealment	Blinding (investigators)	Blinding (participants)	Selective Outcome Reporting	Missing Data	Overall bias
Thase et al.[54] 2015	1	0.5	0.5	1	0.5	0.5	high
Thase et al.[55] 2015	1	0.5	0.5	1	0.5	0.5	high
Theleritis et al.[56] 2017	1	1	0.5	0.5	1	0.5	unclear
Theleritis et al.[56] 2017	1	1	0.5	0.5	1	0.5	unclear
Triggs et al.[57] 2010 (left sham)	0.5	0.5	1	1	1	1	unclear
Triggs et al.[57] 2010 (right sham)	0.5	0.5	1	1	1	1	unclear
Yesavage et al.[61] 2018	1	1	1	1	1	1	low
Zheng et al.[60] 2010	0.5	0	0.5	0.5	1	1	high
Palm et al.[59] 2012	1	1	0.5	1	1	1	low
van Eijndhoven et al.[58] 2020	0.5	0.5	0.5	0.5	1	0	unclear
Rush et al.[79] 2005	1	0.5	1	1	1	0	high
Baeken et al.[80] 2013	0	0.5	0	1	1	1	high
Padberg et al.[78] 1999	0.5	0.5	0.5	1	1	1	unclear

© 2021 Jones BDM et al. *JAMA Network Open*

eTable 2. Subgroup analysis of treatment modalities

Treatment Modality	B Coefficient and 95% CI	p value
Pill		**Ref.**
sham rTMS	-0.24 (-0.53 to 0.05)	0.11
Sham tDCS	0.16 (-0.49 to 0.81)	0.63
Invasive brain stimulation	-0.21 (-0.64 to 0.22)	0.33
Parenteral	0.21 (-0.36 to 0.78)	0.47
Liquid	-0.7 (-1.61 to 2.12)	0.13

© 2021 Jones BDM et al. *JAMA Network Open*

eTable 3. Clinical characteristics

Author and year	Current Episode Length Mean (months)(SD)	Number of Past Episodes (mean) (SD)	Number of Trials Current Episode (mean) (SD)
Bakim et al.[31] 2012	17.7 (9.49)	5 (2.6)	3.3 (1.1)
Barbee et al.[32] 2011	31.8 (43.7)	3.9 (4.6)	
Bauer et al.[75] 2018	8.9 (13.2)	3.4 (2.3)	
Bennabi et al.[74] 2015	NA	NA	NA
Berman et al.[73] 2007	43.6 (53.8)	NA	2.4 (0.6)
Berman et al.[33] 2009	17.2 (NA)	6.3 (10)	2.3 (0.6)
Blumberger et al.[35] 2012	NA	3.8 (3.7)	
Blumberger et al.[34] 2012	40.8 (36)	3.8 (3.7)	4.1 (2.2)
Blumberger et al.[36] 2016	46.9 (110.3)	5.7 (6.7)	
Boutros et al.[37] 2002			
Chen et al.[38] 2013			
Coenen et al.[72] 2019	96 (84)		5.4 (4.9)
Concerto et al.[39] 2015	17.2 (2.7)	4.9 (0.9)	
Dougherty et al.[40] 2015	141.6 (67.2)	2.9 (2)	
Fava et al.[77] 2015			
Fava et al.[41] 2018	31.9 (62.1)		
Fitzgerald et al.[42] 2012		2.8 (2.8)	4.9 (2.6)
Garcia-Toro et al.[76] 2001			
Garcia-Toro et al.[43] 2006	32.6 (26.6)	5.6 (4.4)	
Heresco-Levy et al.[44] 2012	13.2 (14.3)	3 (1.1)	
Hobart et al.[45] 2018	11.7 (14.6)	3.2 (2)	2.2 (0.5)
Hobart et al.[71] 2018	19.4 (46.8)	3.2 (2.4)	2.2 (0.5)
Holtzheimer et al.[46] 2004			
Holtzheimer et al.[70] 2017	116.0 (56.0)	4.4 (3.6)	
Husain et al.[69] 2017			
Ionescu et al.[68] 2019	91.6 (126.4)	5.4 (5.2)	8.2 (3.1)
Kamijima et al.[66] 2013	15.6 (16.4)		2.5 (0.7)
Kamijima et al.[67] 2018	15.8 (35.6)		2.4 (0.6)
Kauffmann et al.[47] 2004			
Li et al.[48] 2014			
Marcus et al.[49] 2008	48.5 (88.8)	7.3 (15.2)	2.4 (0.6)

© 2021 Jones BDM et al. *JAMA Network Open*

Supplementary table 3. continued

Author and year	Current Episode Length Mean (months)(SD)	Number of Past Episodes (mean) (SD)	Number of Trials Current Episode (mean) (SD)
Nierenberg et al.[64] 2003	84.5 (94.9)	0.9 (1.2)	3.5 (2.6)
Palhano-Fontes et al.[63] 2019	10.1 (9.15)	3.5 (1.8)	3.8 (1.9)
Pallanti et al.[51] 2010	9.44 (2.17)	4.8 (2.7)	6.0 (1.7)
Santos et al.[52] 2008	44.5 (66.1)	6.9 (6.9)	
Shelton et al.[53] 2001			
Su et al.[62] 2017			
Thase et al.[54] 2015	13.7 (17.1)	3.8 (2.9)	
Thase et al.[55] 2015	16.9 (35)	3.7 (4.9)	2.24<br>0.467
Theleritis et al.[56] 2017			
Theleritis et al.[56] 2017			
Triggs et al.[57] 2010 (left sham)			
Triggs et al.[57] 2010 (right sham)	.		
Yesavage et al.[61] 2018			
Zheng et al.[60] 2010	54 (32.4)		
Palm et al.[59] 2012	9 (15)		
van Eijndhoven et al.[58] 2020	57.9 (54.8)	3.5 (1.3)	
Rush et al.[79] 2005	51.7 (52.2)		3.4 (1.7)
Baeken et al.[80] 2013	5.3 (4.5)		
Padberg et al.[78] 1999	48 (48)		

© 2021 Jones BDM et al. *JAMA Network Open*

eAppendix 1. Components of the Randomized Effect model used for our analyses

The full statistic description of the Random-effect model and meta-analysis estimation methods used in Stata 17 can be found online on: https://www.stata.com/manuals/meta.pdf

© 2021 Jones BDM et al. *JAMA Network Open*

eAppendix 2. Search Strategy:

Searches were conducted on MEDLINE Web of Science and PsychInfo from inception to January 24th 2020. Key terms, notable papers, and citation lists were reviewed for additional studies. Search terms used were (depress* OR MDD OR major depress*) AND (resistan* OR refractor* OR non-respon* OR nonrespon* OR un-respon* OR unrespon* OR TRD OR fail* OR inadequate OR difficult OR intractable) AND (placebo OR sham OR control OR controlled) AND (randomi* OR RCT) AND (treatment OR intervention OR trial).

© 2021 Jones BDM et al. *JAMA Network Open*

eFigure 1. Funnel plot of included studies

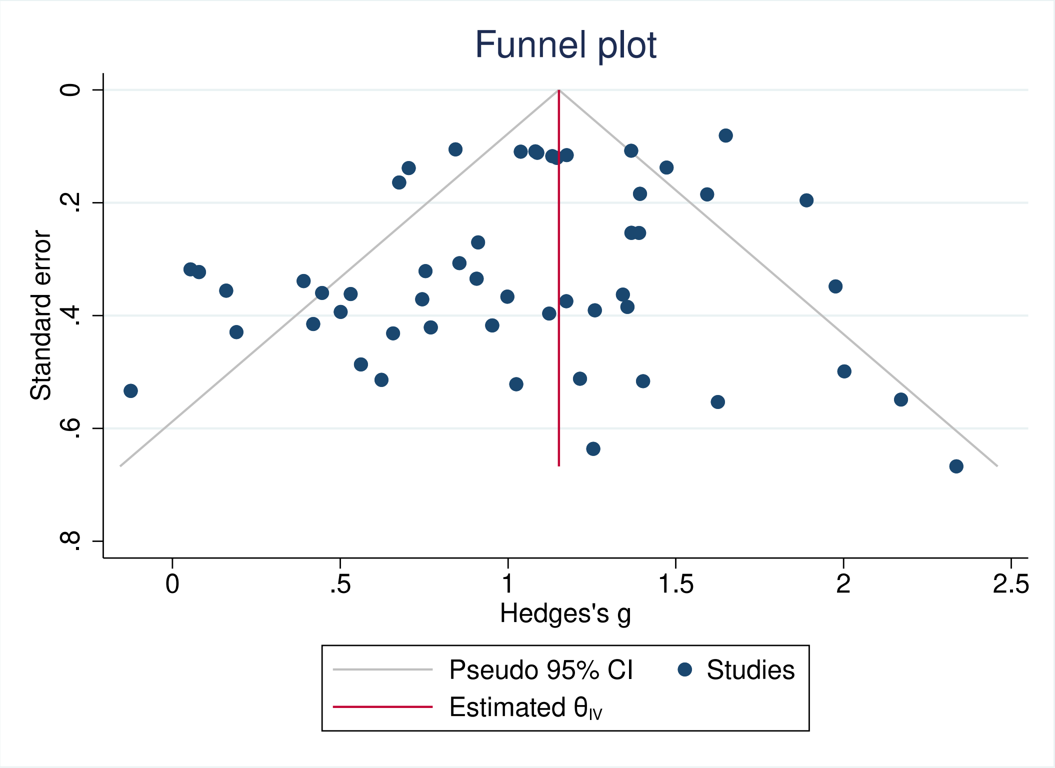

© 2021 Jones BDM et al. *JAMA Network Open*

eFigure 2. Trim and fill funnel plot

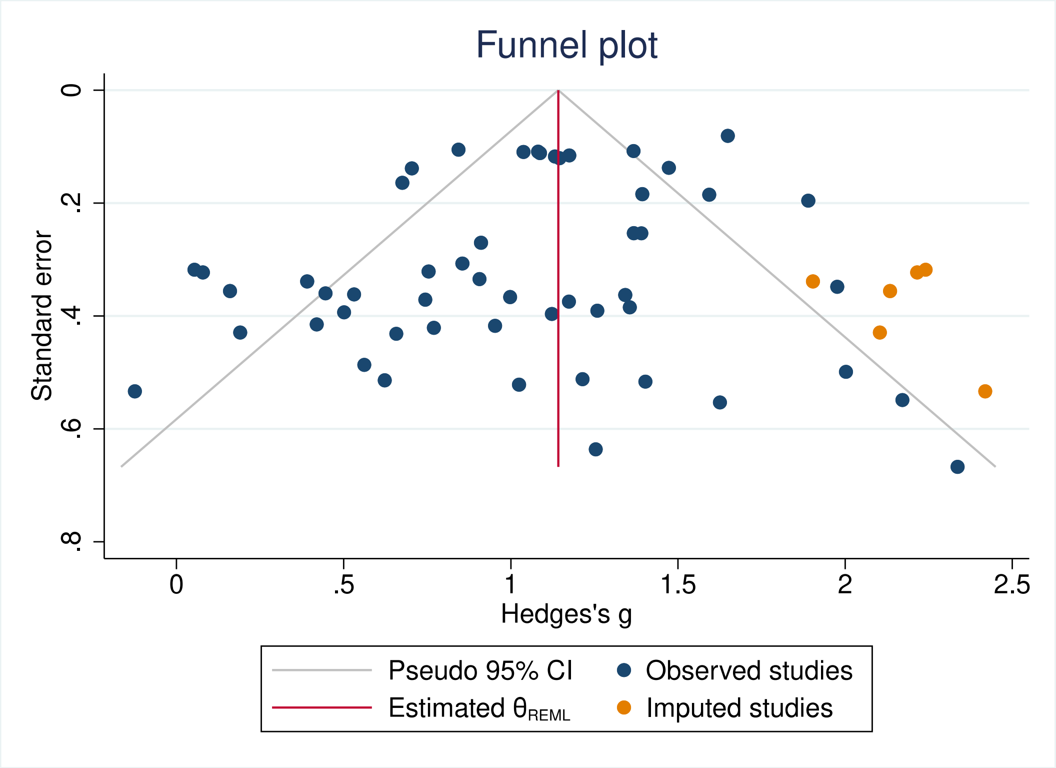

© 2021 Jones BDM et al. *JAMA Network Open*

eFigure 3. Forest plot of main placebo effect size Grouped by risk of bias

Study	Baseline N	Baseline Mean	Baseline SD	Endpoint N	Endpoint Mean	Endpoint SD	Hedges's g with 95% CI	Weight (%)
**Low**								
Barbee et al. 2011	48	26.63	4.88	31	17.37	8.62	1.39 [0.89, 1.89]	2.24
Berman et al. 2007	176	25.9	6.5	160	20.1	2.9	1.13 [0.90, 1.36]	3.01
Blumberger et al. 2012	11	24.1	2.9	7	18.1	5.5	1.40 [0.39, 2.41]	1.11
Blumberger et al. 2012	22	25.2	2.8	15	18.9	6.4	1.34 [0.63, 2.05]	1.68
Blumberger et al. 2016	41	25.5	3.6	35	20.5	3.64	1.37 [0.87, 1.86]	2.25
Boutros et al. 2002	9	31.7	4.9	7	26.4	12.4	0.56 [-0.39, 1.52]	1.20
Dougherty et al. 2015	14	36.4	3.3	14	27.4	10.5	1.12 [0.35, 1.90]	1.53
Fava et al. 2018	81	26.4	6.3	72	18.4	2.89	1.59 [1.23, 1.96]	2.64
Heresco-Levy et al. 2012	13	27.2	4.9	12	23.3	9.6	0.50 [-0.27, 1.27]	1.54
Hobart et al. 2018	202	26.2	6.2	96	18.1	3.53	1.47 [1.20, 1.74]	2.91
Holtzheimer et al. 2017	30	37.3	3.8	29	30	10.6	0.91 [0.38, 1.44]	2.15
Ionescu et al. 2019	13	26.3	4.8	10	20	10.7	0.77 [-0.06, 1.59]	1.43
Kamijima et al. 2018	203	25.2	6.5	183	18	6.8	1.08 [0.87, 1.30]	3.05
McAllister-Williams et al. 2015	82	28.1	5.4	74	22.6	10.3	0.68 [0.35, 1.00]	2.76
Palm et al. 2012	11	34.6	5.4	10	30.2	7.4	0.66 [-0.19, 1.50]	1.39
Yesavage et al. 2018	83	27.5	5.1	68	14.4	8.6	1.89 [1.51, 2.27]	2.58
Heterogeneity: $\tau^2 = 0.09$, $I^2 = 67.67\%$, $H^2 = 3.09$							1.18 [0.98, 1.38]	
Test of $\theta_i = \theta_j$: Q(15) = 42.58, p < 0.001								
**Unclear**								
Bakim et al. 2012	12	25.58	3.82	12	19.5	7.83	0.95 [0.13, 1.77]	1.45
Bennabi et al. 2015	12	24.2	5.6	11	13.17	4.97	2.00 [1.02, 2.98]	1.17
Berman et al. 2009	172	27.1	5.8	149	20.7	5.3	1.15 [0.91, 1.38]	2.99
Garcia-Toro et al. 2001	18	25.6	4.92	16	23.83	3.78	0.39 [-0.27, 1.05]	1.79
Garcia-Toro et al. 2006	10	25.1	7.28	10	23.6	7.79	0.19 [-0.65, 1.03]	1.40
Hobart et al. 2018 2	206	25.4	5.2	186	20.8	5.7	0.84 [0.64, 1.05]	3.06
Holtzheimer et al. 2004	8	20.8	6.3	7	15.3	3	1.02 [0.00, 2.05]	1.10
Husain et al. 2017	20	32.6	10.1	18	32	11.8	0.05 [-0.57, 0.68]	1.89
Kamijima et al. 2013	195	25.5	7.4	183	18.1	6.8	1.04 [0.82, 1.25]	3.04
Kauffmann et al. 2004	5	18.2	4.9	5	11.8	4.3	1.25 [0.01, 2.50]	0.83
Li et al. 2014	15	23.8	3.2	15	19.66	3.2	1.26 [0.49, 2.02]	1.56
Nierenberg et al. 2003	17	21.7	4.2	15	15.1	5.3	1.36 [0.60, 2.11]	1.58
Padberg et al. 1999	6	22.2	8.8	6	23.5	10.4	-0.12 [-1.17, 0.92]	1.07
Palhano-Fontes et al. 2019	15	19.73	4.59	15	16.92	7.36	0.45 [-0.26, 1.15]	1.69
Su et al. 2017	24	23.3	4.1	24	13.78	5.3	1.98 [1.29, 2.66]	1.75
Theleritis et al. 2017	20	29.4	3.2	18	25.4	5.3	0.91 [0.25, 1.56]	1.81
Theleritis et al. 2017 2	24	30.3	3.6	21	27	4	0.86 [0.25, 1.46]	1.95
Triggs et al. 2010 (left sham)	7	27.7	3.5	7	22	11.6	0.62 [-0.38, 1.63]	1.12
Triggs et al. 2010 (right sham)	7	27.3	2.7	7	13.4	7.4	2.34 [1.03, 3.64]	0.77
Heterogeneity: $\tau^2 = 0.16$, $I^2 = 71.12\%$, $H^2 = 3.46$							0.94 [0.70, 1.18]	
Test of $\theta_i = \theta_j$: Q(18) = 44.00, p < 0.001								
**High**								
Baeken et al. 2013	11	26.45	8.71	11	22.36	10.01	0.42 [-0.39, 1.23]	1.46
Bauer et al. 2018	441	25.8	4.1	381	12.41	11.07	1.65 [1.49, 1.81]	3.16
Chen et al. 2013	10	24.9	6.3	10	12.3	4.7	2.17 [1.10, 3.25]	1.03
Coenen et al. 2019	8	29.6	4	8	21.46	5.37	1.63 [0.54, 2.71]	1.01
Concerto et al. 2015	15	21	5	15	20	7	0.16 [-0.54, 0.86]	1.71
Fava et al. 2015	85	29.1	5.5	63	20.8	6.46	1.39 [1.03, 1.75]	2.65
Fitzgerald et al. 2012	20	22.9	2.1	17	22.6	5	0.08 [-0.55, 0.71]	1.87
Marcus et al. 2008	190	27	5.5	162	21.3	3.93	1.17 [0.95, 1.40]	3.02
Mu-Hong Chen et al. 2019	16	34	4.7	16	26.1	8	1.17 [0.44, 1.91]	1.63
Pallanti et al. 2010	20	29.05	3.54	20	26.38	3.4	0.75 [0.12, 1.38]	1.88
Rush et al. 2005	110	29.7	5.2	110	25.1	7.6	0.70 [0.43, 0.98]	2.90
Santos et al. 2008	17	28.4	7.7	13	21.2	11.3	0.74 [0.02, 1.47]	1.64
Shelton et al. 2001	10	27.7	4.2	7	20.8	6.8	1.21 [0.21, 2.22]	1.13
Thase et al. 2015 1	191	27.1	5.6	178	21.95	3.56	1.09 [0.87, 1.31]	3.03
Thase et al. 2015 2	221	26.3	5.3	203	20	3.69	1.37 [1.16, 1.58]	3.05
Zheng et al. 2010	15	24.6	2.8	15	22.9	3.4	0.53 [-0.18, 1.24]	1.68
van Eijndhoven et al. 2020	16	22.7	3.8	16	18.6	4.2	1.00 [0.28, 1.72]	1.66
Heterogeneity: $\tau^2 = 0.16$, $I^2 = 82.32\%$, $H^2 = 5.66$							1.02 [0.78, 1.26]	
Test of $\theta_i = \theta_j$: Q(16) = 82.63, p < 0.001								
**Overall**							1.05 [0.91, 1.18]	
Heterogeneity: $\tau^2 = 0.14$, $I^2 = 76.19\%$, $H^2 = 4.20$								
Test of $\theta_i = \theta_j$: Q(51) = 185.86, p < 0.001								
Test of group differences: $Q_b(2) = 2.34$, p = 0.31								

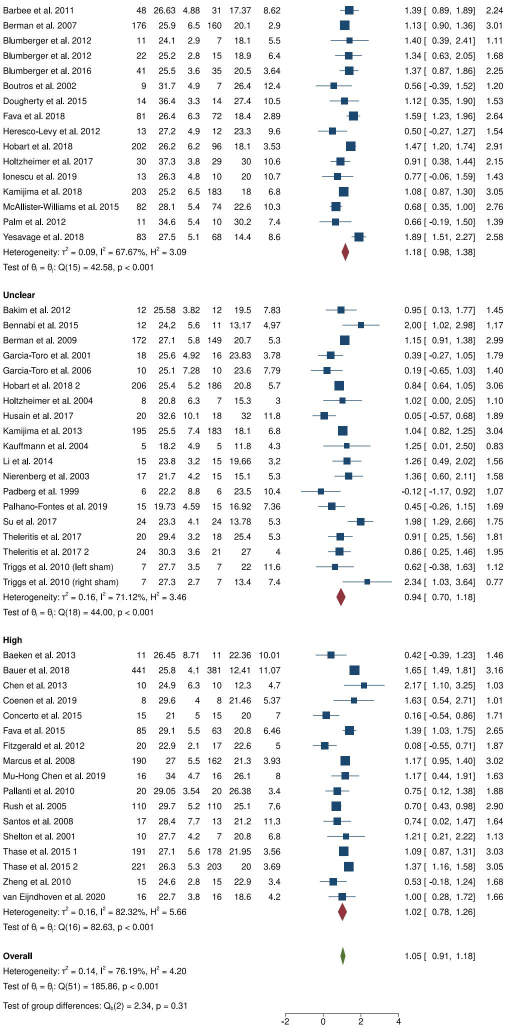

Random-effects REML model

© 2021 Jones BDM et al. *JAMA Network Open*
